# Increasing airflow ventilation in a nasal maxillary ostium using optimised shape and pulsating flows

**DOI:** 10.1007/s10237-025-01971-6

**Published:** 2025-06-25

**Authors:** Patrick Warfield-McAlpine, David F. Fletcher, Fiona Zhang, Kiao Inthavong

**Affiliations:** 1 Department of Mechanical Manufacturing Mechatronic Engineering, RMIT University, PO Box 71, Melbourne, Victoria 3000 Australia; 2 School of Chemical and Biomolecular Engineering, The University of Sydney, Sydney, NSW 2006 Australia

**Keywords:** Nasal airway, CFD, Pulsating flow, T-junction, Laminar

## Abstract

Ventilation of the maxillary sinus is essential for regulating pressure, preventing infection and providing mucous to the nasal anatomy. During infection, the pathway between the sinus and the nasal airway (ostia) can become inflamed and restrict ventilation. Surgery is often required to restore airflow. The current surgical standard involves the widening of the ostium. Although this restores fluid flow, it has been linked to post-surgical sequelae. This study examined the effects of pulsating flow and geometric modifications on airflow distribution in a T-junction model analogous to a nasal maxillary ostium. A circular T-junction with variable anterior and posterior radius of curvature ($R_c$) was used to simulate airflow through the nasal maxillary ostium, investigating flow behaviour under oscillatory inlet velocities at frequencies of 30, 45, 60, and 75 Hz. Computational fluid dynamics (CFD) simulations assessed how flow distribution through the nasal cavity and maxillary ostium (represented by the *x*- and *y*-branches) is affected by curvature and oscillatory frequency, focusing on implications for respiratory airflow, particle delivery and inhalation toxicology. Results indicated that increasing the anterior $R_c$ enhanced airflow into the *y*-branch (analogous to the maxillary ostium), while posterior curvature had minimal impact. Higher oscillatory frequencies increased reverse flow, which may improve ventilation but could interfere with consistent drug delivery. These insights are valuable for optimising respiratory therapies and inhalation toxicology.

## Introduction

T-junctions are integral components across various engineering disciplines, playing a significant role in fluid flow management, mixing, and distribution in many applications. While T-junctions can be used to merge and mix fluids, they can also divert a fluid in different directions. For example, industrial applications, including HVAC systems, split and distribute air to rooms of a building and piping systems distribute water, oil or gas to specific locations. However, in this study, we focus on a T-junction representing the human nasal ostium airway for distributing air to the maxillary sinuses that sit laterally to the main nasal passage.

**Fig. 1:**
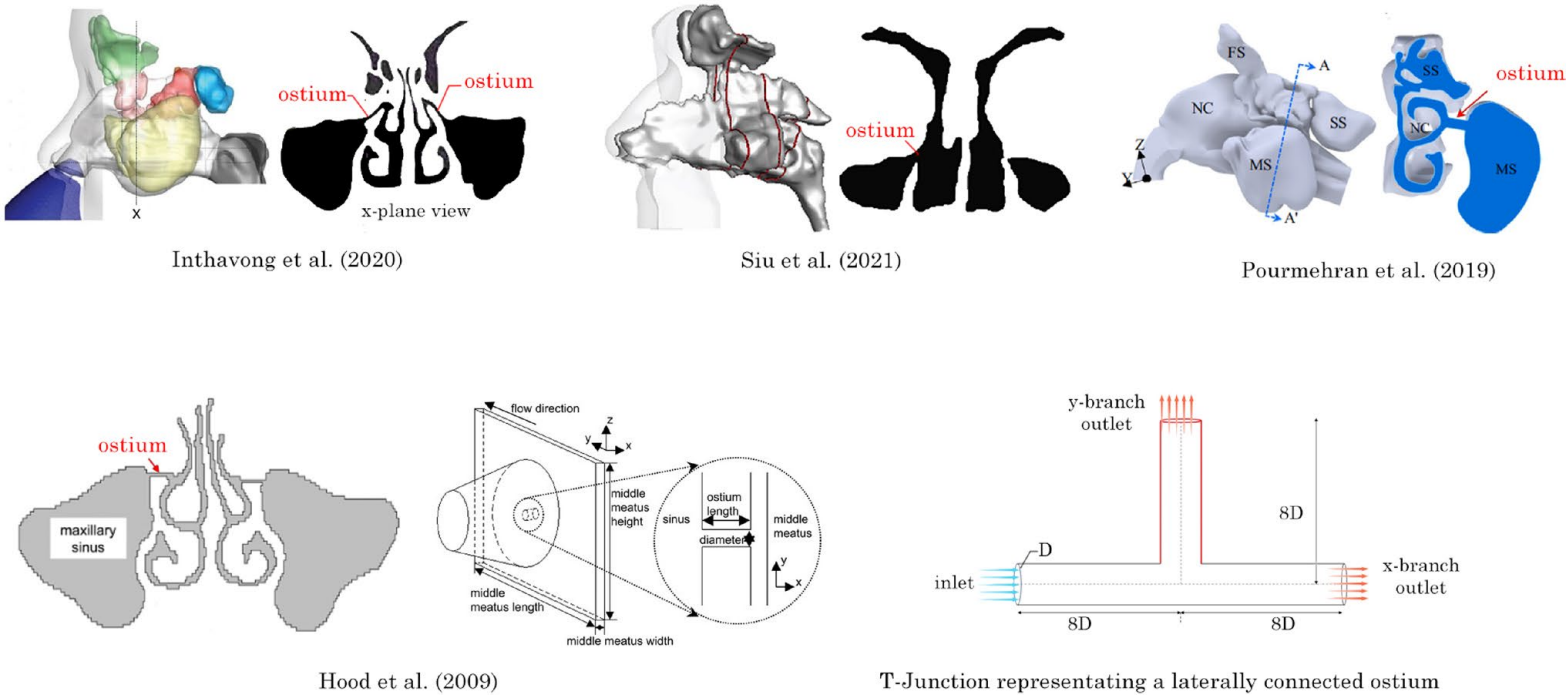
Diverging flows of T-junction geometries found at the maxillary ostium

The narrow, laterally positioned ostium hinders effective ventilation and drug delivery to sinus-related conditions. Figure 1 shows various CFD studies of the nasal cavity that depict the maxillary ostia (Inthavong et al. 2020; Pourmehran et al. 2020; Hood et al. 2009; Siu et al. 2021) where the ostia can be identified as a long cylindrical tube connecting the nasal airway to the maxillary sinus. This tube can be approximated as a T-junction geometry to explore the effects of modifying the ostium anatomy in a generalised manner. This approach is similar to the representative rectangular plane and T-junction geometry used by Hood et al. (2009).

The maxillary sinuses are the largest of the paranasal sinuses and function as the main source of mucous to lubricate the nasal airway and condition inhaled air for effective oxygen exchange in the lungs. Inflammation in the maxillary sinus can lead to blockage of the paranasal sinuses’ drainage pathway (ostia), which impairs ventilation and mucociliary clearance. When this occurs, the airway needs to be decongested via medical therapy using a course of antibiotics or corticosteroid nasal sprays (Bose et al. 2016) to restore sinus ventilation and allow drainage.

However, significant challenges prevent effective treatment due to the airway geometry with the maxillary sinus sitting laterally to the airflow direction (Prasanna and Mamatha 2010). The maxillary sinus is connected to the nasal airway by the ostia, a narrow tubular channel, measuring approximately 1 to 5 mm in diameter and spanning from 1 to 7 mm in length (Nouraei et al. 2009; El-Anwar et al. 2018). When medical therapy fails to fully decongest the maxillary sinus, surgery is required to remove the diseased tissue and restore ventilation.

Functional endoscopic sinus surgery (FESS) opens the obstructed sinus openings by removing ostia tissue, to improve sinus ventilation and restore mucociliary clearance (Anselmo-Lima et al. 2007; Khalil and Nunez 2006). This is generally achieved by increasing the size of the ostium, subsequently providing additional volume for fluid transport. As the nasal anatomy varies considerably, the required enlargement differs between patients. Consequently, patient-specific planning based on medical imagery is essential. The enlargement size can play a significant role in patient comfort. Kirihene et al. (2002) showed an enlargement of the maxillary sinus ostium above its normal size (20 $\hbox {mm}^2$) produced a significant decrease in both the maxillary sinus and the nasal cavity nitrous oxide levels; a chemical compound essential for host defence.

In addition to surgery, patients continue with topical drug delivery to target the inflamed mucosa of the sinus (Rudmik et al. 2011). Optimising airflow dynamics within the nasal cavity is crucial for effectively delivering drugs and enhancing treatment results. Delivering drugs to treat the symptoms of chronic rhinosinusitis is challenging due to poor ventilation and limited access to the ostia.

Grobler et al. (2008) assessed the maxillary penetration of a nasally administered saline douche using blue dye on 17 patients with variable ostium diameters. This study found that diameters <1.26 mm had no blue dye penetration and that a minimum diameter of 3.95 mm was required to guarantee a 95% probability of penetration. As the average range of ostium diameters is from 1 to 5 mm, this study suggests that nasal douching is not a suitable treatment for all patients and that patients with ostium diameters <3.95 mm may benefit from an enlargement of the ostium prior to post-surgical medical therapy.

As *in vivo* and *in vitro* studies are time-consuming, researchers have used Computational Fluid Dynamics (CFD) to identify nasal airflow physiology. Early research assessing the flow characteristics of the human nasal cavity largely focused on time-independent solutions (steady-state); however, the realisation that respiratory airflow is inherently unsteady led to a shift towards studying time-dependent (transient) phenomena. Doorly et al. (2008) used (CFD) to simulate the transient flow during breathing cycles, revealing the complex nature of the flow patterns. This research was further built on by Bradshaw et al. (2022) using a high-fidelity hybrid turbulence model that explored the transient effects of fluid flow on air conditioning of the inhaled air.

The impact of pulsating flow in T-junctions has gained significant research interest due to its potential to enhance airflow penetration and mixing. For instance, Khodadadi et al. (1988) presented LDA measurements and numerical predictions of pulsatile laminar flow in a two-dimensional $\hbox {90}^\circ$ bifurcated channel. They found that pulsation frequency and amplitude introduced additional complexity to flow patterns, such as affecting separation zones and flow reattachment points. Higher Reynolds numbers further contributed to larger recirculation regions. Selimefendigil et al. (2021) extended this work to investigate heat transfer under pulsating laminar flow in a $\hbox {90}^\circ$ bifurcation, revealing that more fluid was directed towards the *y*-branch, increasing thermal fluctuations.

Studies assessing the effect of pulsatile flow on drug delivery within the nasal cavity identified enhanced particle dispersion and deposition. Möller et al. (2008) applied a pulsating flow *in vivo* at 45 Hz and found a 2-4% increase in paranasal sinus deposition and three times the deposition in the nasal airway. Maniscalco et al. (2006), reported comparable findings and showed enhanced deposition in the nasal cavity as the frequency was raised.

Pulsatile flow has also been studied for its effects on drug delivery in the nasal cavity, where the flow geometry is not necessarily a T-junction. For example, Moeller et al. (2009) applied pulsating flow *in vivo* at 45 Hz and observed that 3-5% of nasally delivered aerosols deposited in the paranasal sinus, along with a threefold increase in deposition in the nasal airway. Similarly, Maniscalco et al. (2006) reported enhanced deposition with higher pulsation frequencies. In addition, Farnoud et al. (2020) explored the effect of varying delivery angles ($\hbox {45}^\circ$ and $\hbox {90}^\circ$), finding that a $\hbox {45}^\circ$ angle combined with pulsating flow at 45 Hz increased flow partitioning into the maxillary sinus by 0.03% and deposition in the nasal airway by 44.6%. Hosseini and Golshahi (2019) further analysed the effects of a pulsating nebuliser in airway models across different age groups (2, 5, and 50 years), concluding that a frequency of 44.5 Hz increased maxillary sinus deposition. Additionally, Pourmehran et al. (2020, 2021) investigated the use of acoustic waves in nasal nebulisers, demonstrating that acoustic amplitude, initial velocity, and particle size enhanced aerosol deposition.

By studying a T-junction geometry and its impact on airflow distribution, we aim to determine the optimal surgical strategy-based airflow penetration into the ostium anatomy from the nasal cavity to enhance ventilation and drug delivery to the paranasal sinuses. The T-junction diverts inhaled fluid into two outlets; one outlet represents the perpendicular maxillary ostium, and the other represents the continuous remaining nasal airway. A parametric study is performed using a range of anterior and posterior radius of curvature ($R_c$) at the T-junction. Pulsating inlet velocity conditions with frequencies of 30, 45, 60, and 75 Hz were used. This corresponds to current nebuliser drug delivery devices, which can identify optimal conditions for enhancing fluid penetration and ventilation into the maxillary sinus.

## Method

### Geometry models

**Fig. 2:**
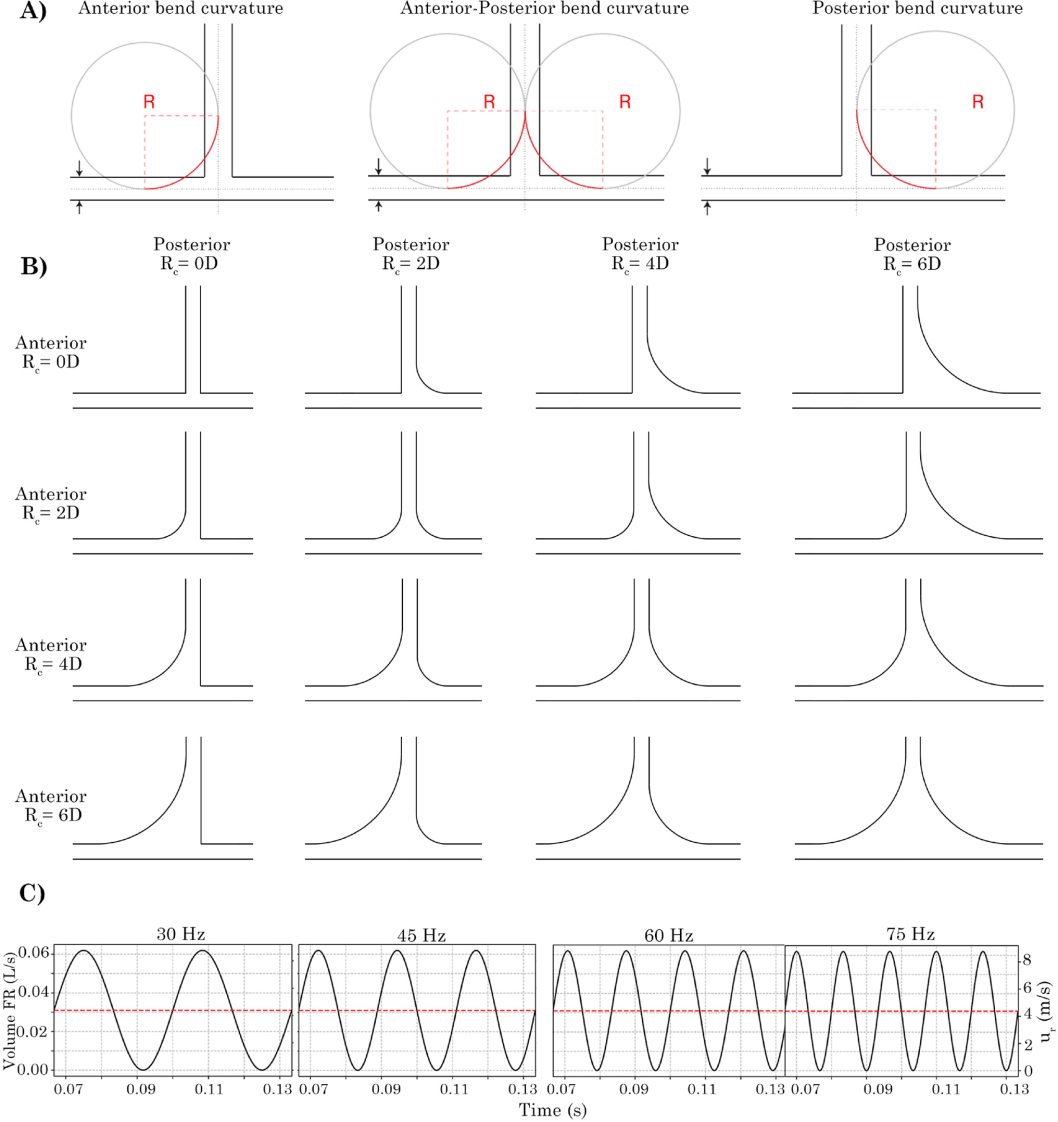
Schematic depicting the method for producing bend curvature onto the T-junction geometry. Radial bend curvatures of 2*D*, 4*D* and 6*D* (and 0*D* for no curvature) were used. 3D geometry models depicting the anterior curvature, both anterior-posterior curvature and posterior-only curvature. Four oscillatory flow given by $u(r,t) = u_{r}(1 + \sin (2\pi f t ))$ applied at the velocity inlet are shown for the time range of t = 0.0667 to 0.1334 s

The circular T-junction was modelled in Ansys SpaceClaim (v2024R1) with an internal diameter of $D=3$ mm. The entry and exit branch lengths were defined as 8*D* in the *x*-branch, and 8*D* in the *y*-branch, to minimise reverse flow at the exits. The $R_c$ on the anterior and posterior sides of the junction were varied to assess the enhanced airflow that could exit through the *y*-branch; see Fig. 2. Using $R_c$ of 0*D*, 2*D*, 4*D* and 6*D*, sixteen models from combinations of the anterior and posterior alterations were produced.

**Fig. 3:**
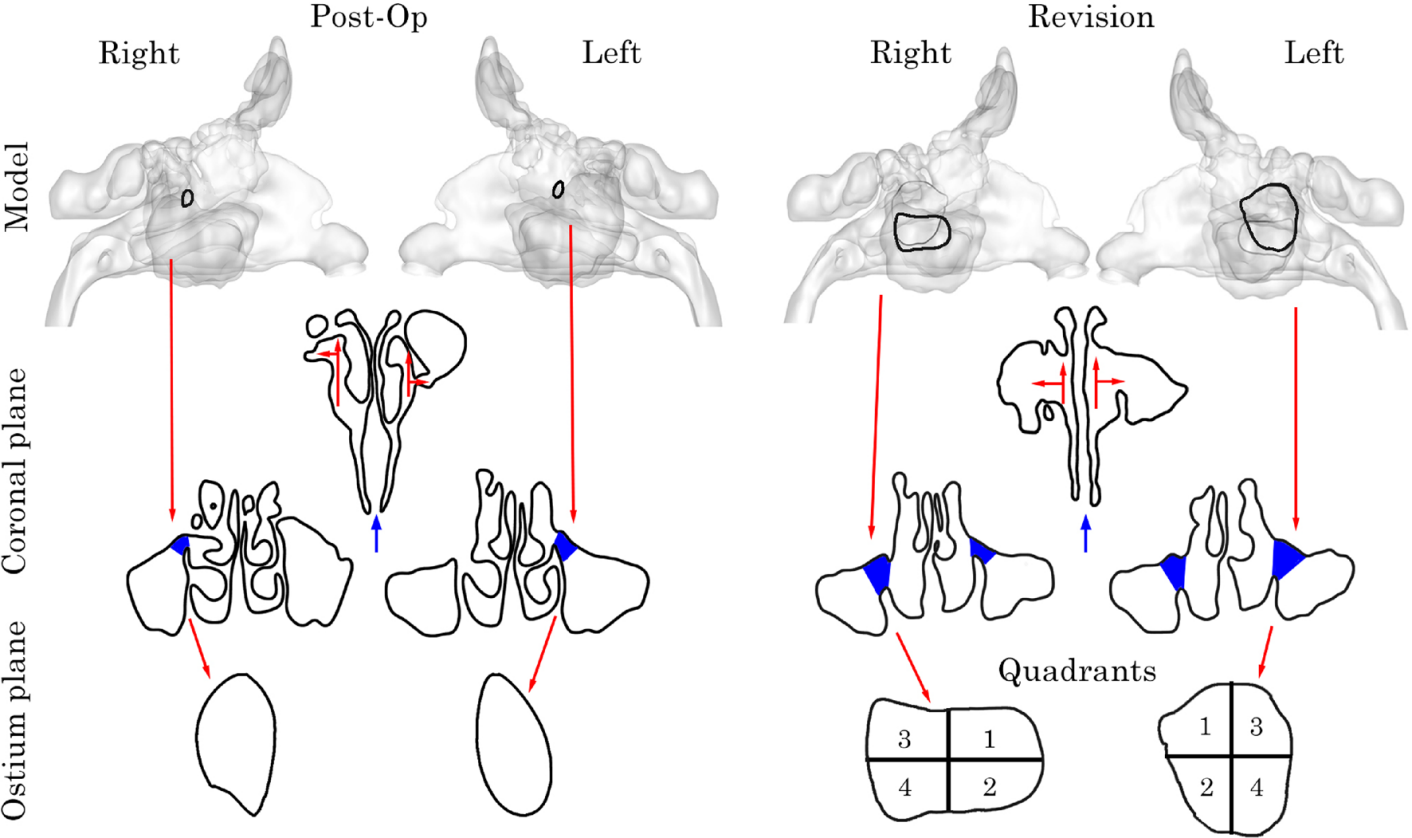
Post-Op and Revision surgery geometry models. Coronal plane taken at centre of ostium inlet highlighting T-junction analogue. Left and right ostium inlet planes. Revision model separated into 4 quadrants to better capture flow characteristics due to its large size

The nasal airway models were generated from CT scans of a single female patient age 66 yr that had undergone two comprehensive FESS procedures. The surgical procedures are labelled as Post-operative (Post-Op) and Revision, see later, in Fig. 3. The nasal vestibules of both models were capped and extended by 1 mm to create a planar surface to act as the velocity inlet boundary condition and the nasopharynx was extended to improve numerical convergence.

### Transient and oscillatory flow settings

A fully developed laminar flow profile was applied at the pipe geometry inlet to generate steady fluid flow defined as:

1 $$\begin{aligned} u_{r} = 2u_{avg}\left( 1- \left( \frac{r}{R} \right) ^2 \right) \end{aligned}$$

where $u_{avg}$ is the average bulk flow velocity, *r* is the radial coordinate from the pipe centre and *R* is the pipe radius.

This profile was then multiplied by a sinusoidal function to produce oscillatory flow under transient conditions using the following equation:

2 $$\begin{aligned} u(r,t) = u_{r}(1 + \sin (2\pi f t )) \end{aligned}$$

where $u_{r}$ is the laminar velocity profile, *f* is the frequency of oscillation and *t* is the flow time. Four oscillating frequencies, $f=$ 30 Hz, 45 Hz, 60 Hz, and 75 Hz, were investigated, and their profiles as a function of time are shown in Fig. 2c. The velocity profiles are shown for the time range of 0.1332 s to 0.2664 s, which correspond to the time period used for analysis and represent the lowest common multiple range for complete cycles for all four frequencies. Limiting the analysis to this period also avoids start-up effects influencing the results from $t=0$ s to $t=0.1332$ s. A time step of 1$\times 10 ^{-5}$ s was used for all cases which was associated with the mesh grid size to capture the unsteady laminar flow and maintain a $\textrm{CFL}$ (Courant-Friedrichs-Lewy) number of $\sim$1 for all frequencies. Table 1 summarises the unsteady simulation settings for the four frequencies and the associated Womersley and Strouhal numbers.

**Table 1:** Unsteady simulation settings, together with Womersley ($W_o$) and Strouhal (*St*) numbers

Frequency (Hz)	Period (s)	Cycles start-up	Cycles analysed	Flow time (s)	$W_o$	*St*
30	0.0333	2	2	0.1333	10.8	0.006
45	0.0222	3	3	0.1333	13.2	0.009
60	0.0167	4	4	0.1333	15.2	0.012
75	0.0133	5	5	0.1333	17.0	0.015

The Womersley number ($W_o$) is a dimensionless parameter that relates the pulsating frequency to the viscous effects of the flow simulation for scalability, given by:

3 $$\begin{aligned} W_o = D\sqrt{\frac{\omega \rho }{\mu }} \end{aligned}$$

where *D* is the diameter of the pipe, $\omega$ is the angular frequency of the oscillation, $\rho$ is the density, and $\mu$ is the dynamic viscosity of the fluid. When the $W_o$ is greater than one, the pulsating frequency is a significant parameter to consider and the flow cannot be estimated using a quasi-steady approach.

The Strouhal number (*St*) is used to characterise oscillatory flows. It is defined as the ratio of the frequency of vortex shedding to the mean flow velocity and is calculated by:

4 $$\begin{aligned} St = \frac{f D}{v} \end{aligned}$$

where *f* is the oscillation frequency, *D* is the pipe diameter and *v* is the average fluid velocity.

At high *St* numbers, flow is dominated by oscillations, whereas at low *St*, the impact of oscillations on fluid flow is minimal. Under standard breathing conditions, the *St* is typically low, implying that oscillatory effects are negligible. However, since this study applies higher inlet oscillation frequencies, *St* must be taken into account to ensure that oscillatory effects are properly captured.

A sinusoidal velocity boundary condition is applied, where a velocity of 15 m/s represents the peak of the sinusoidal wave. The ($W_o$) calculated at each frequency are high, indicating that the flow is significantly influenced by oscillations. As a result, the flow cannot be approximated using a quasi-steady approach, and transient conditions are necessary to accurately estimate the flow field.

Although the *St* numbers associated with each frequency are low, suggesting that vortex shedding at a velocity of 15 m/s will be negligible, the sinusoidal velocity condition introduces velocity fluctuations. These fluctuations induce unsteady laminar behaviour, which requires the simulation to have sufficient time resolution to capture this dynamic phenomenon.

The nasal models were first simulated under steady-state conditions at a constant volumetric-flow rate of 7.5 L/min at each nostril inlet associated with a total resting inhalation flow rate of 15 L/min, and a 0 gauge pressure outlet following the nasopharynx extension. Following the steady-state simulation the area-weighted average velocity was exported from the inlet of each nostril surface and applied to the oscillatory flow equation, Equ. (5).

5 $$\begin{aligned} u(avg,t) = u_{avg}(1 + \sin (2\pi f t )) \end{aligned}$$

**Fig. 4:**
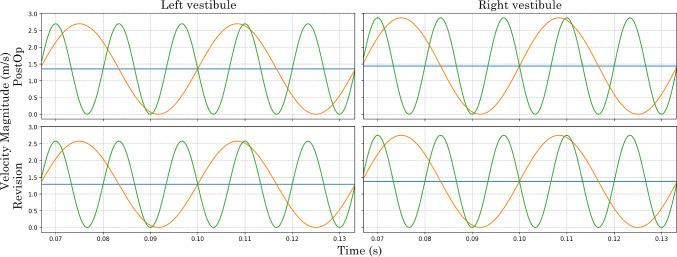
Velocity magnitude at the nasal vestibule inlets recorded for Post-Op and Revision model from 0.0667$-$0.1333 s

Following simulation the velocity magnitude was recorded at the nasal vestibule inlets to demonstrate the pulsating flow condition, Fig. 4.

### Meshing

**Fig. 5:**
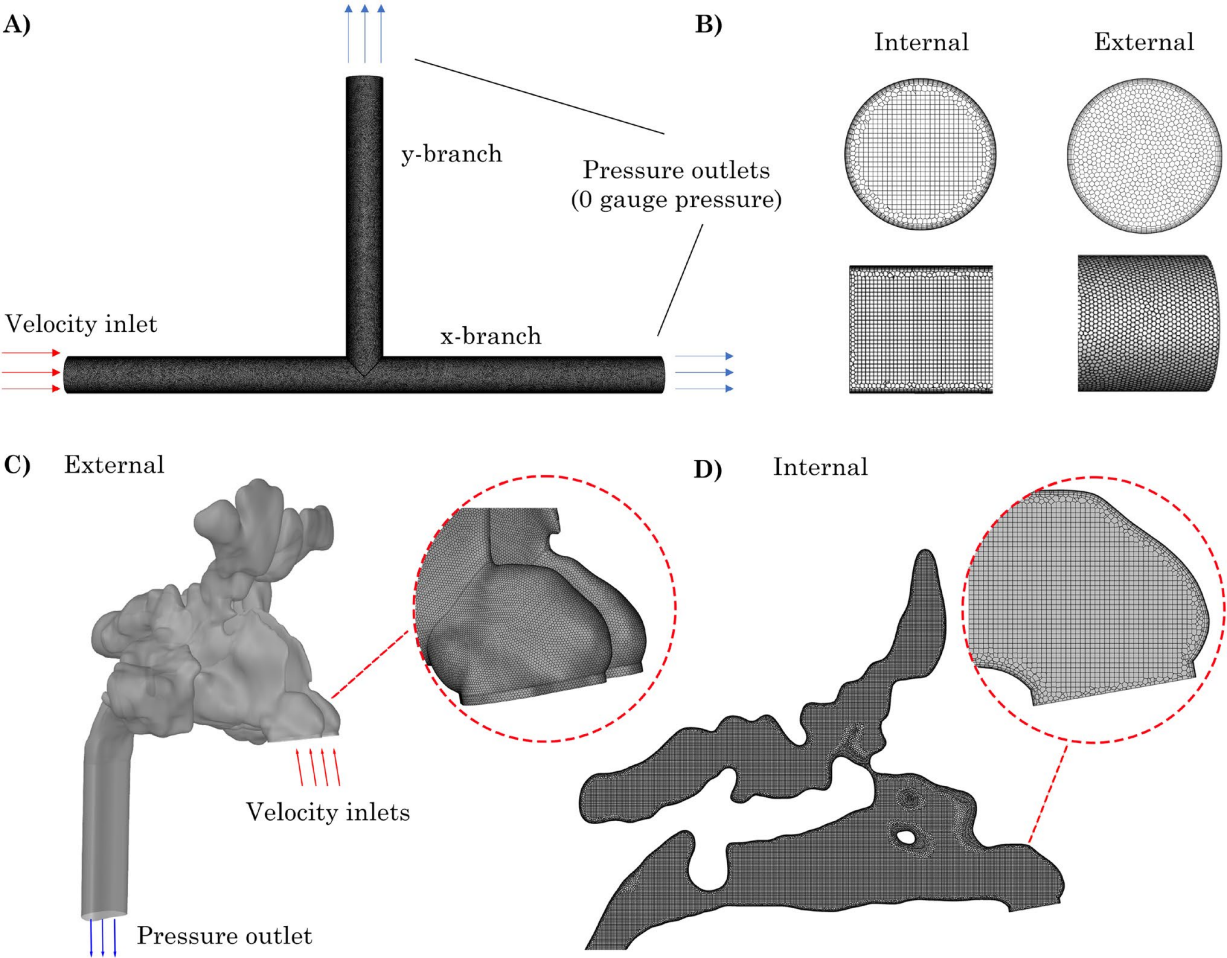
A) Sample geometry from standard T-junction model with no anterior or exterior radius of curvature (0*D* $\times$ 0*D*), B) Internal hexahedral and external polyhedral mesh elements. C) External mesh and boundary conditions for Revision nasal model. D) Internal hexcore mesh for Revision model

T-junction and airway models were meshed using Ansys Fluent Meshing (v2024R1) with poly-hexcore cell configurations, where the surface boundaries were polyhedrals and the internal cells were hexahedrals, (Fig. 5).

**Fig. 6:**
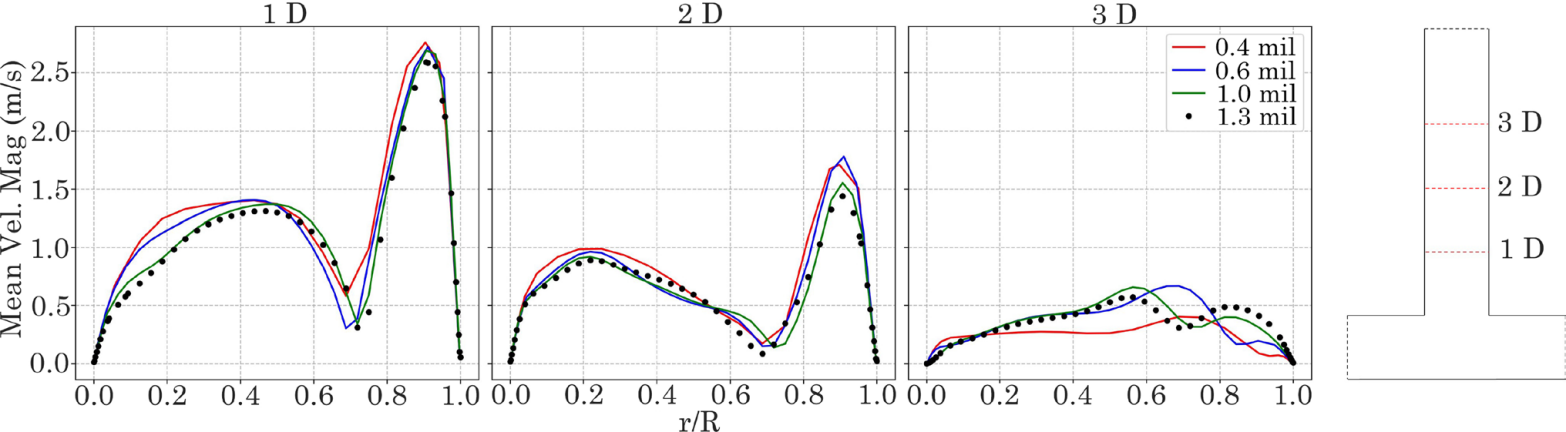
Normalised velocity profiles at reattachment point 1 D, 2 D and 3 D in ostium exit length. Coarse: 400,000, Medium = 600,000, Fine: 1,000,000, and Very Fine: 1,300,000 elements

Four T-junction meshed models were created and evaluated for mesh independence, and Table 2 summarises the mesh settings. An unsteady laminar flow with Reynolds number of Re = 1800 (associated with the peak flow during flow pulsation) was used. Velocity profiles at 1D, 2D, and 3D from the *y*-branch were plotted for all mesh densities (Fig. 6) and the results showed that a mesh density > 1 million cells was required for mesh independence.

**Table 2:** Summary of different mesh resolution parameters for the T-junction models, where $N_{cells}$ is the number of mesh cells/elements, $\Delta$ is the grid size, $N_{pl}$ is the number of prism layers, and $h_{pl}$ is the first prism layer height

Mesh	$N_{cells}\times 10^6$	$\Delta$ (mm)	$N_{pl}$	$h_{pl}$ (mm)
Mesh 1	0.40	0.150	6	0.006
Mesh 2	0.60	0.145	6	0.006
Mesh 3	1.00	0.100	6	0.006
Mesh 4	1.30	0.080	6	0.006

Given the complexity of nasal geometries, the fault-tolerant workflow in Ansys Fluent (v2024R1) was employed to wrap surfaces and eliminate leakage from segmentation. Element sizing methods included a body of influence of 0.2 mm for internal cells, curvature on surfaces and edges, and proximity on all surfaces with a maximum and minimum size of 8 mm and 0.4 mm, respectively. Six prism layers were added to the fluid wall regions using the last-ratio specification, with a first cell height of 0.004 mm and an transition ratio of 0.26. Additionally, two peel layers were implemented to ensure a smooth transition between polyhedral and hexahedral elements. The final mesh had a total element count of 5 million. Following the methodology presented in Inthavong et al. (2018), mesh independence was achieved. The models have previously been published in Siu et al. (2020, 2021).

### CFD modelling

Whilst Re remains below the transitional limit (Re $\le$ 1800), a $W_o \le 17$ suggests that the flow is pulsatile and inertia-dominated, with viscous effects confined to near-wall regions. A study by Xu et al. (2017), investigated the transition to turbulence during pulsatile flow in smooth pipes and found that under the applied conditions the flow can be approximated as quasi-laminar. Subsequently, transient simulations were performed for laminar flow for all models. The continuity and momentum equations of an incompressible fluid are given as:

6 $$\begin{aligned} & \nabla \cdot (\vec {u}) = 0 \end{aligned}$$

7 $$\begin{aligned} & \frac{\partial }{\partial t}(\rho \vec {u}) + \nabla \cdot (\rho \vec {u}\otimes \vec {u}) = - \nabla p + \nabla \cdot (\bar{\bar{\tau }}) \end{aligned}$$

where $\bar{\bar{\tau }}$ is the stress tensor given by:

8 $$\begin{aligned} \bar{\bar{\tau }} = \mu \left[ (\nabla \vec {u} + \nabla \vec {u}^T) \right] \end{aligned}$$

where $\mu$ is the molecular viscosity and *I* is the unit tensor. Note in this application the second term is zero because the flow is incompressible.

Normal and axial outlets were set to zero gauge pressure. The equations were solved using Ansys Fluent (v2024R1). Spatial discretisation for pressure and momentum used second-order and second-order upwind schemes, respectively, while flow gradients within cells used the least squares cell-based method. The SIMPLE algorithm was used for the pressure–velocity coupling. The simulations were deemed converged when the local residual error fell below 1$\times 10^{-5}$ for continuity and momentum. Oscillatory frequencies of 30 Hz, 45 Hz, 60 Hz and 75 Hz were applied to generate pulsating flow using Eqn. (2) and Eqn. (5), in the T-junction models and nasal cavity models, respectively.

## Results

### Oscillatory flow in the baseline T-Junction model

A baseline simulation was first conducted using the model with $R_c$ of 0*D* $\times$ 0*D* (denoting the anterior $\times$ posterior radii of curvature). The simulation was run at each frequency for a flow time of 0.0667 s to establish a developed flow field (after transient start-up cycles, see Table 1). Thereafter, data were recorded between $t = 0.0667$ to 0.1333 s, and the average volumetric-flow rate (L/min) at the *x*- and *y*-branch outlets were recorded at each time step.

The input wave flow profile first accelerates flow through the *x*-branch, creating a low-pressure region at the T-junction due to the negative pressure gradient, which causes reverse or suction flow from the *y*-branch. As the input flow decelerates, the pressure increases, and the flow exits through the *y*-branch outlet. In both scenarios, the pulsating inlet flow causes a delayed response. At the *x*-branch outlet, this delay in volume flow rate results from the phase shift of the input signal as it propagates downstream.

Table 3 summarises the response delay at the outlets given in units of degrees (360 per cycle) for the baseline case of 0*D* $\times$ 0*D*, and one extreme of each anterior and posterior curvatures of $R_c=6D$, e.g. 0*D* $\times$ 6*D*, and 6*D* $\times$ 0*D*, respectively. For the baseline case, the response delay was short at the *x*-branch outlet, and this increased as the input frequency increased, ranging from $\hbox {2.7}^\circ$ to $\hbox {4.5}^\circ$. At the *y*-branch outlet, the response delay was much longer and was generally unaffected by an increase in the input frequency, ranging from $\hbox {25.2}^\circ$ to $\hbox {25.7}^\circ$.

The response delay at the *x*-branch outlet increases with higher oscillatory frequencies and anterior $R_c$. However, in the posterior curved model (0*D* $\times$ 6*D*), an 18% decrease in response delay is observed between 60 Hz and 75 Hz (Table 3). Additionally, the response delay at the *x*-branch outlet is significantly smaller than at the *y*-branch outlet, as expected, since most of the fluid exits through the *x*-branch outlet.

**Table 3:** Response delay, in phase angle ($^\circ$), for the flow rates through the *x*-branch and *y*-branch outlets

Freq. Hz	*x*-branch			*y*-branch		
	0*D* $\times$ 0*D*	0*D* $\times$ 6*D*	6*D* $\times$ 0*D*	0*D* $\times$ 0*D*	0*D* $\times$ 6*D*	6*D* $\times$ 0*D*
30	$\hbox {2.7}^\circ$	$\hbox {3.9}^\circ$	$\hbox {4.3}^\circ$	$\hbox {25.7}^\circ$	$\hbox {25.2}^\circ$	$\hbox {25.6}^\circ$
45	$\hbox {3.4}^\circ$	$\hbox {4.6}^\circ$	$\hbox {6.9}^\circ$	$\hbox {25.2}^\circ$	$\hbox {24.2}^\circ$	$\hbox {25.3}^\circ$
60	$\hbox {4.0}^\circ$	$\hbox {5.1}^\circ$	$\hbox {7.5}^\circ$	$\hbox {25.7}^\circ$	$\hbox {25.8}^\circ$	$\hbox {26.7}^\circ$
75	$\hbox {4.5}^\circ$	$\hbox {4.5}^\circ$	$\hbox {7.7}^\circ$	$\hbox {25.5}^\circ$	$\hbox {26.8}^\circ$	$\hbox {26.9}^\circ$

### Velocity profiles in the T-junction branches

**Fig. 7:**
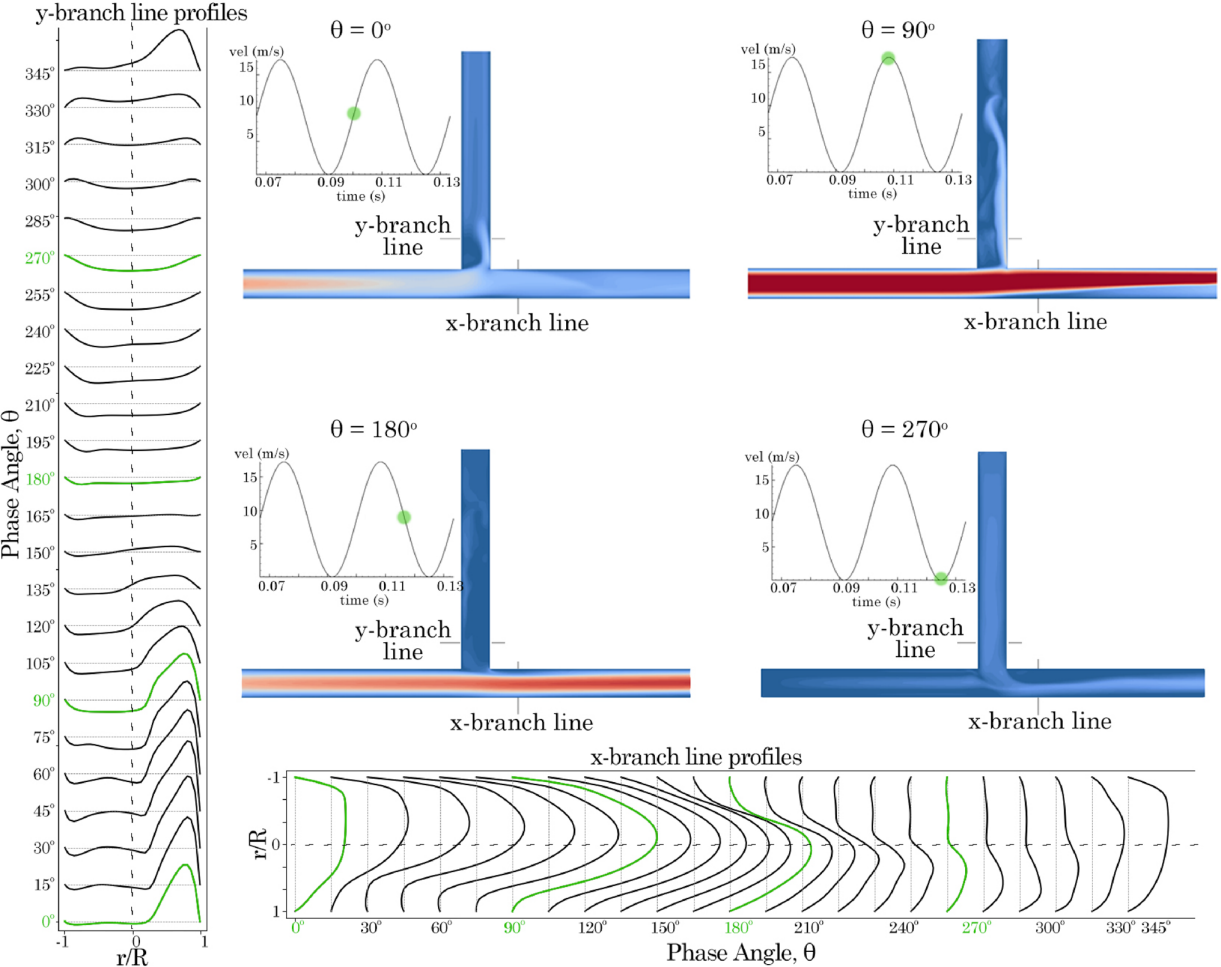
Velocity profiles 1 D from the T-junction in the *x*- and *y*-branches for model 0*D* $\times$ 0*D* varying with time at 30 Hz. Contours are plotted at $\hbox {0}^{\circ }$, $\hbox {90}^{\circ }$, $\hbox {180}^{\circ }$, and $\hbox {270}^{\circ }$ phase angles

**Fig. 8:**
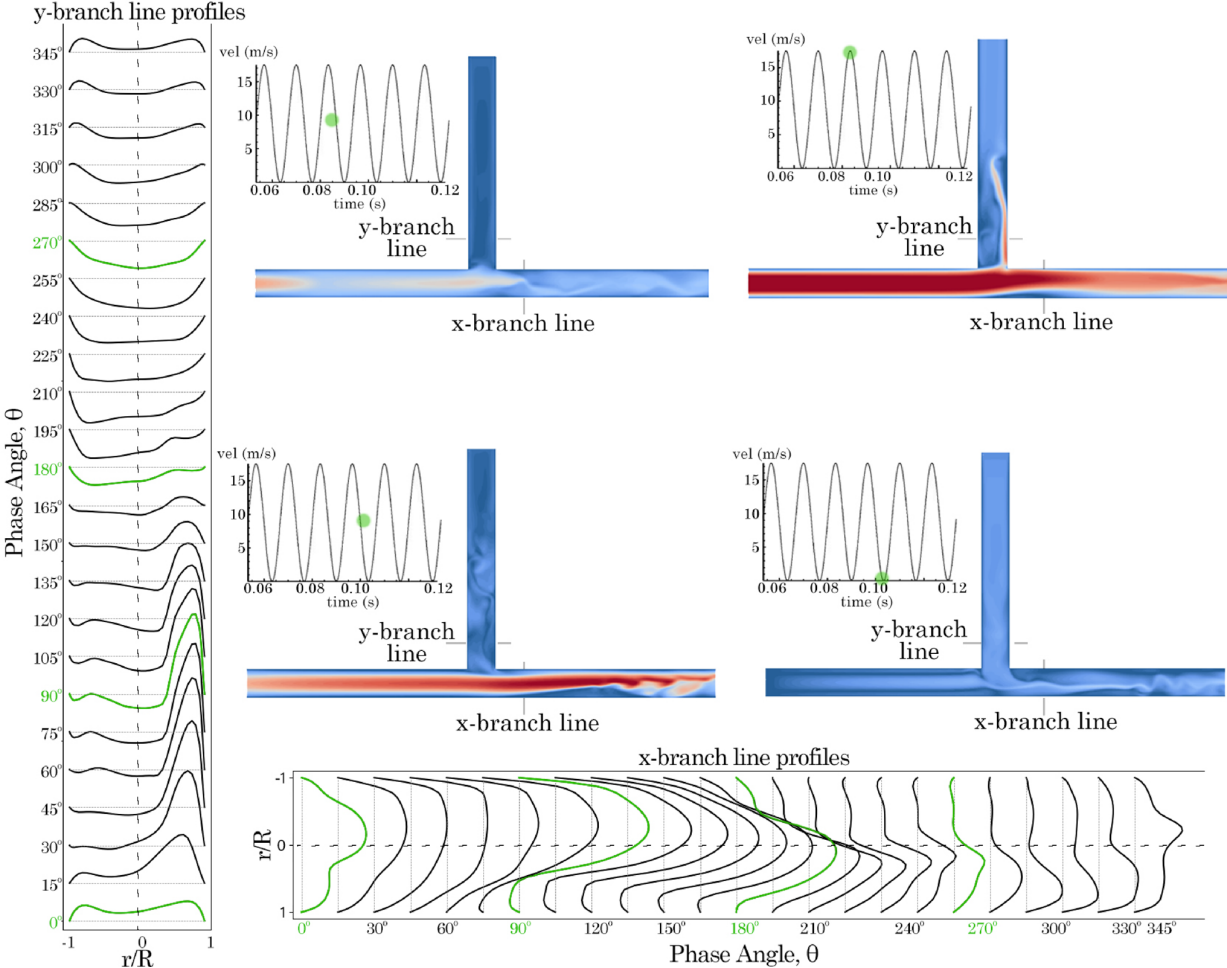
Velocity profiles 1 D from the T-junction in the *x*- and *y*-branches for model 0*D* $\times$ 0*D* varying with time at 75 Hz. Contours are plotted at $\hbox {0}^{\circ }$, $\hbox {90}^{\circ }$, $\hbox {180}^{\circ }$, and $\hbox {270}^{\circ }$ phase angles

Velocity line profiles were plotted at $\hbox {15}^{\circ }$ phase angle intervals at 1 D downstream of the T-junction (denoted as *x*-branch and *y*-branch lines) for frequencies of 30 Hz (Fig. 7) and 75 Hz, (Fig. 8). At 30 Hz in the *y*-branch, exit flow occurred from $\hbox {0}^{\circ }$ to $\hbox {150}^{\circ }$, with the velocity profile skewed towards the posterior side (where $r/R \rightarrow 1$). Flow direction reversed between $\hbox {165}^{\circ }$ and $\hbox {300}^{\circ }$, producing a more parabolic velocity profile due to suction. Exit flow resumed for the remainder of the cycle.

In the *x*-branch, only exit flow was observed throughout the period. Initially, at $\hbox {0}^{\circ }$, the velocity profile exhibited a skewed parabolic shape, which increased in magnitude and became more symmetrical as it approached $\hbox {180}^{\circ }$. However, as suction occurred in the *y*-branch, the velocity magnitude decreased, and the profile skewed towards the lower wall. When the frequency was increased to 75 Hz, the results were similar but with more pronounced asymmetry in the skewed profiles. In the *x*-branch, suction from the *y*-branch led to increased unsteadiness after $\hbox {180}^{\circ }$.

**Fig. 9:**
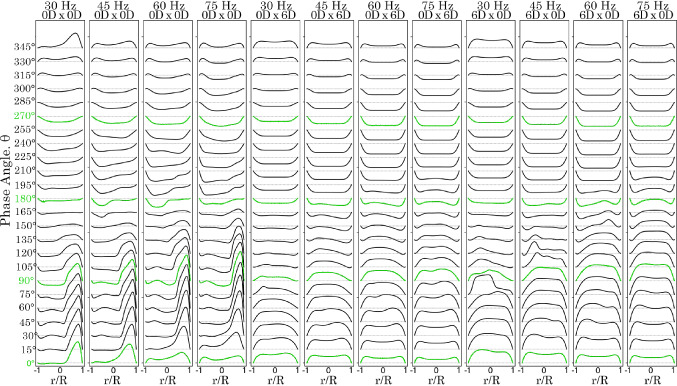
Velocity profiles for time at $\hbox {15}^\circ$ intervals, 1 D from the T-junction on the *y*-branch length for models 0*D* $\times$ 0*D*, 0*D* $\times$ 6*D* and 6*D* $\times$ 0

**Fig. 10:**
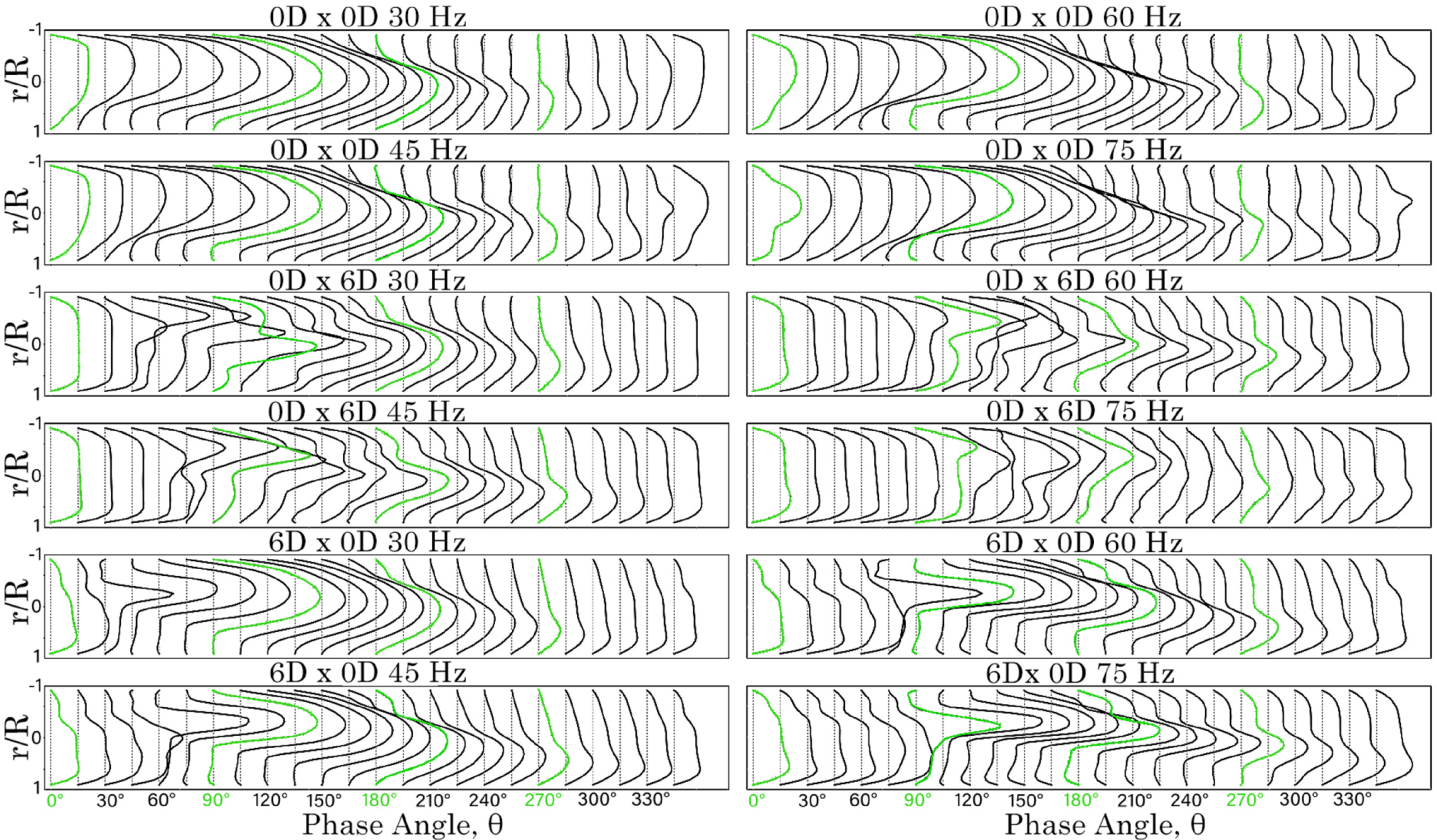
Velocity profiles for time at $\hbox {15}^\circ$ intervals, 1 D from the T-junction on the *x*-branch length for models 0D $\times$ 0D, 0D $\times$ 6*D* and 6*D* $\times$ 0*D*

A comparison of the velocity profiles for the 0*D* $\times$ 0*D*, 0*D* $\times$ 6*D*, and 6*D* $\times$ 0*D* models under all frequency conditions is shown in Figs. 9 and 10. In the 0*D* $\times$ 0*D* model, the peak velocity at the posterior wall of the *y*-branch increases as the frequency rises. However, for the 0*D* $\times$ 6*D* and 6*D* $\times$ 0*D* models, the curved bends eliminate the sudden flow separation observed in the 0*D* $\times$ 0*D* model at the T-junction regions. The anterior $R_c = 6D$ model exhibited a higher velocity magnitude in the positive exit direction from $\hbox {0}^{\circ }$ to $\hbox {90}^{\circ }$. However, the reverse suction flow velocity profiles from $\hbox {180}^{\circ }$ to $\hbox {345}^{\circ }$ were comparable between both the anterior and posterior $R_c = 6D$ models.

In the *x*-branch, the posterior $R_c$ model (0*D* $\times$ 6*D*), resulted in greater mixing, leading to more irregular velocity profiles. In contrast, the anterior $R_c$ model 6*D* $\times$ 0*D* produced smoother, more parabolic velocity profiles. This indicates that with an anterior $R_c = 0$, sharp flow separation is maintained, contributing to downstream flow breakdown. In all cases, the peak velocity intensities occurred between $\hbox {0}^{\circ }$ and $\hbox {90}^{\circ }$ in the *y*-branch, and between $\hbox {90}^{\circ }$ and $\hbox {240}^{\circ }$ in the *x*-branch.

### Velocity contour fields

**Fig. 11:**
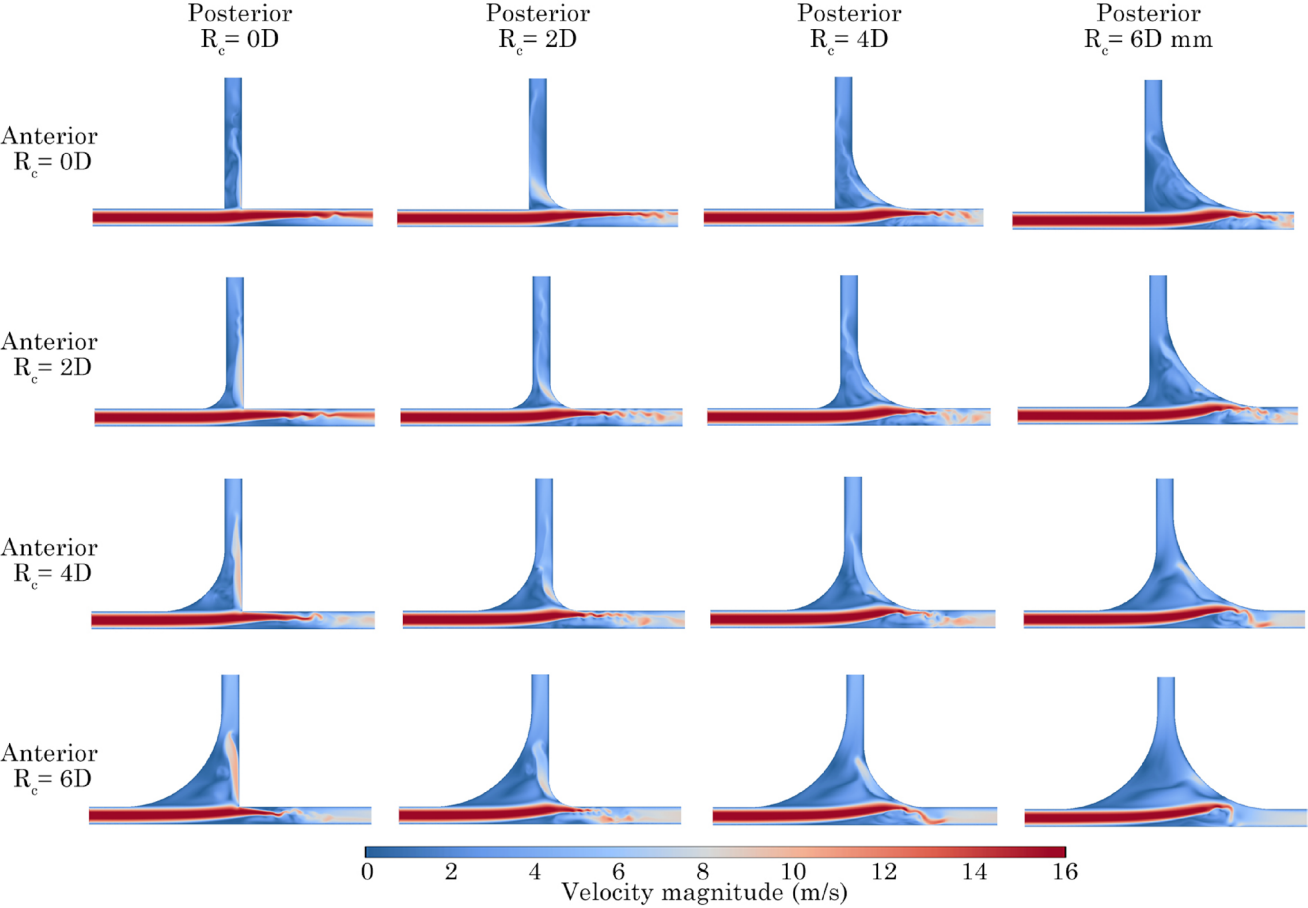
Peak velocity boundary condition magnitude contours at 45 Hz for all configurations. Anterior and posterior $R_c$ are represented by rows and columns, respectively

Velocity contours on the centre plane were exported for all models at the peak inlet velocity at a frequency of 45 Hz (Fig. 11). The highest velocities occur along the *x*-branch, while lower velocities are observed through the *y*-branch. As the flow moves past the T-junction, an increase in velocity is noted in the posterior *y*-branch for models with posterior $R_c$ of 0*D* and 2*D*. As the posterior angle increases,  recirculation occurs, and the bulk flow is redirected towards the *x*-branch. Increasing the anterior $R_c$ further enhances the flow impinging on the posterior *y*-branch wall, resulting in more flow exiting through the *y*-branch outlet.

**Fig. 12:**
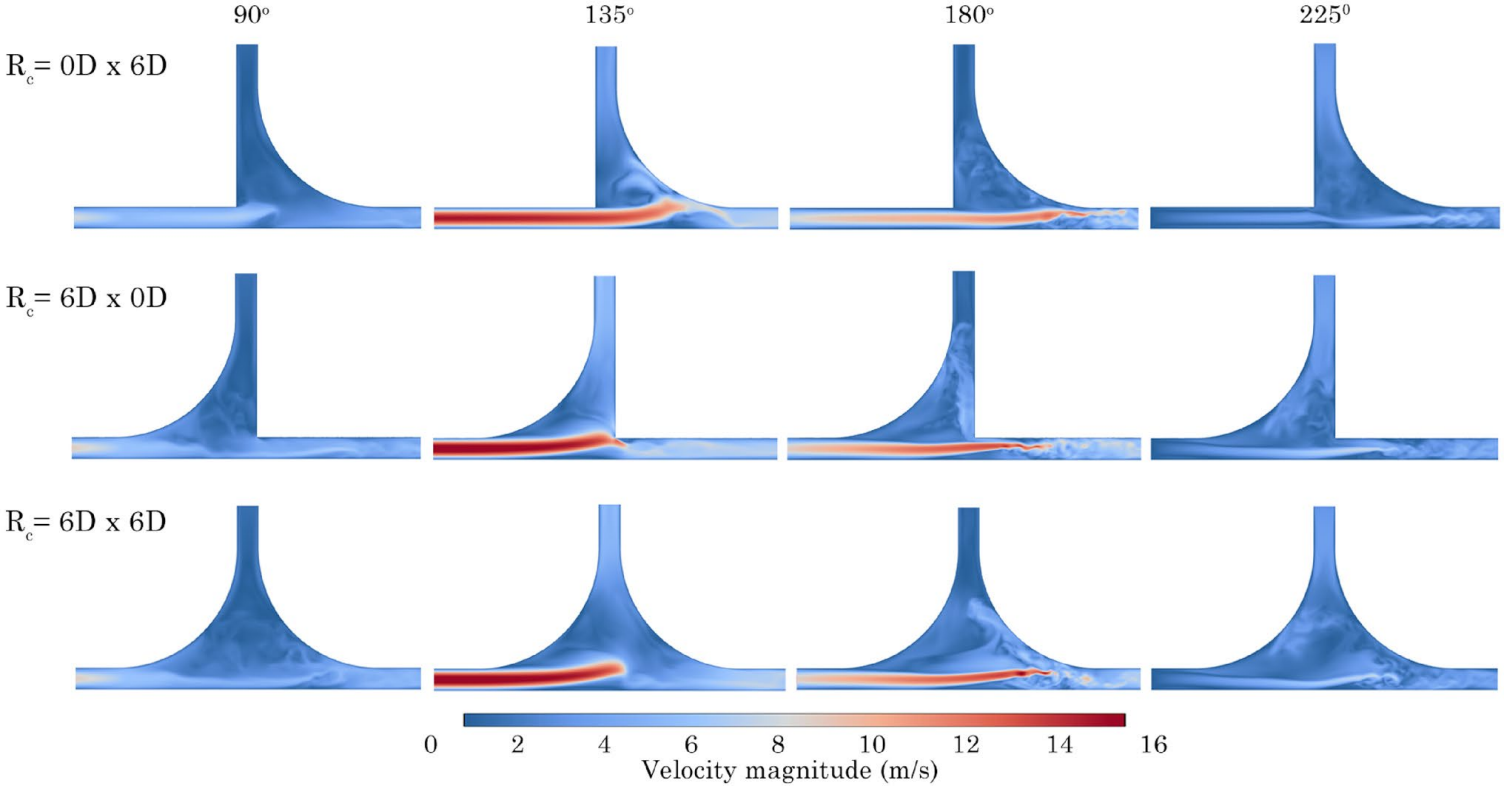
Velocity contours for models 0*D* $\times$ 6*D*, 6*D* $\times$ 0*D* and 6*D* $\times$ 6*D*, at phase angles $\hbox {90}^{\circ }$, $\hbox {135}^{\circ }$, $\hbox {180}^{\circ }$, $\hbox {225}^{\circ }$ at 75 Hz

To understand the effect of the pulsating flow boundary condition, contours were extracted at key phase angles, ($\hbox {90}^{\circ }$, $\hbox {180}^{\circ }$, $\hbox {135}^{\circ }$, $\hbox {225}^{\circ }$) for models 0*D* $\times$ 6*D*, 6*D* $\times$ 0*D* and 6*D* $\times$ 6*D* at 75 Hz, Fig. 12. The addition of $R_c$ increased the cross-sectional area before the T-junction, which led to a decrease in fluid momentum. Figure 12 exhibits higher velocities in the *x*-branch, with minimal fluid flow directed towards the *y*-branch outlet at all stages of development. In contrast, the 6*D* $\times$ 0*D* model experienced fluid impaction on the posterior wall, causing fluid to be redirected towards the *y*-branch.

Maximising both the anterior and posterior $R_c$ (6*D* $\times$ 6*D*) significantly reduced the fluid velocity through the T-junction. As a result, only a minimal amount of fluid was directed towards the *y*-branch, and a recirculation pattern developed within the T-junction, Fig. 12.

### Steady volumetric-flow rates through the branches

**Fig. 13:**
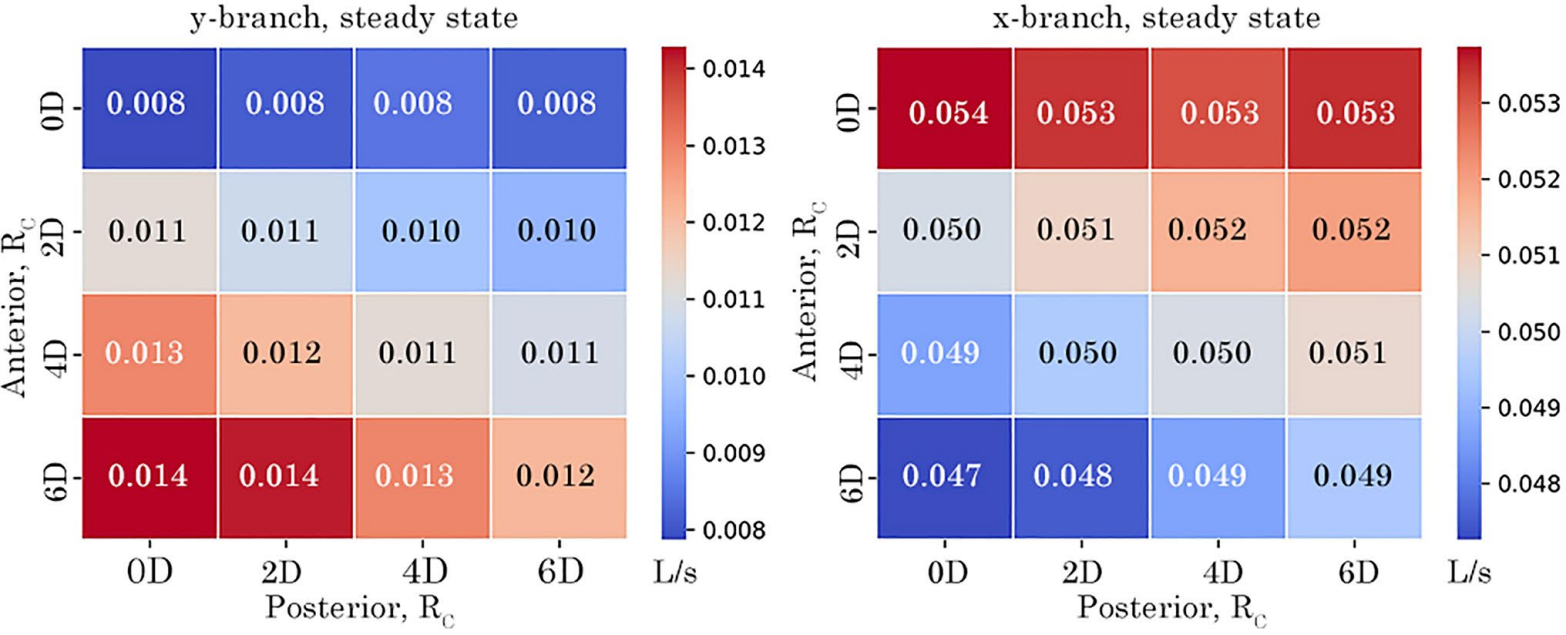
Steady volumetric-flow rate at *y*- and *x*-branch outlet comparing the effect of anterior and posterior $R_c$ on volumetric outflow

A baseline steady-state simulation was performed to compare the volumetric-flow rate through the *x*- and *y*-branches. The results for all models are plotted as a heat map in Fig. 13 to show minimum and maximum values. Modifying the anterior $R_c$ had the most significant effect on the *y*-branch flow. As the anterior $R_c$ increased, the exit flow also increased, reaching a maximum value of 0.84 L/min for the 6*D* $\times$ 0*D* model. Conversely, increasing the posterior $R_c$ reduced the *y*-branch flow across all configurations. In the *x*-branch, decreasing the anterior $R_c$ led to an increase in flow, whereas changes in the posterior $R_c$ had minimal impact. The *x*-branch exhibited higher flow overall, approximately 3.3 to 6.8 times greater than the *y*-branch.

### Oscillatory flow penetration through the branches for all configurations

**Fig. 14:**
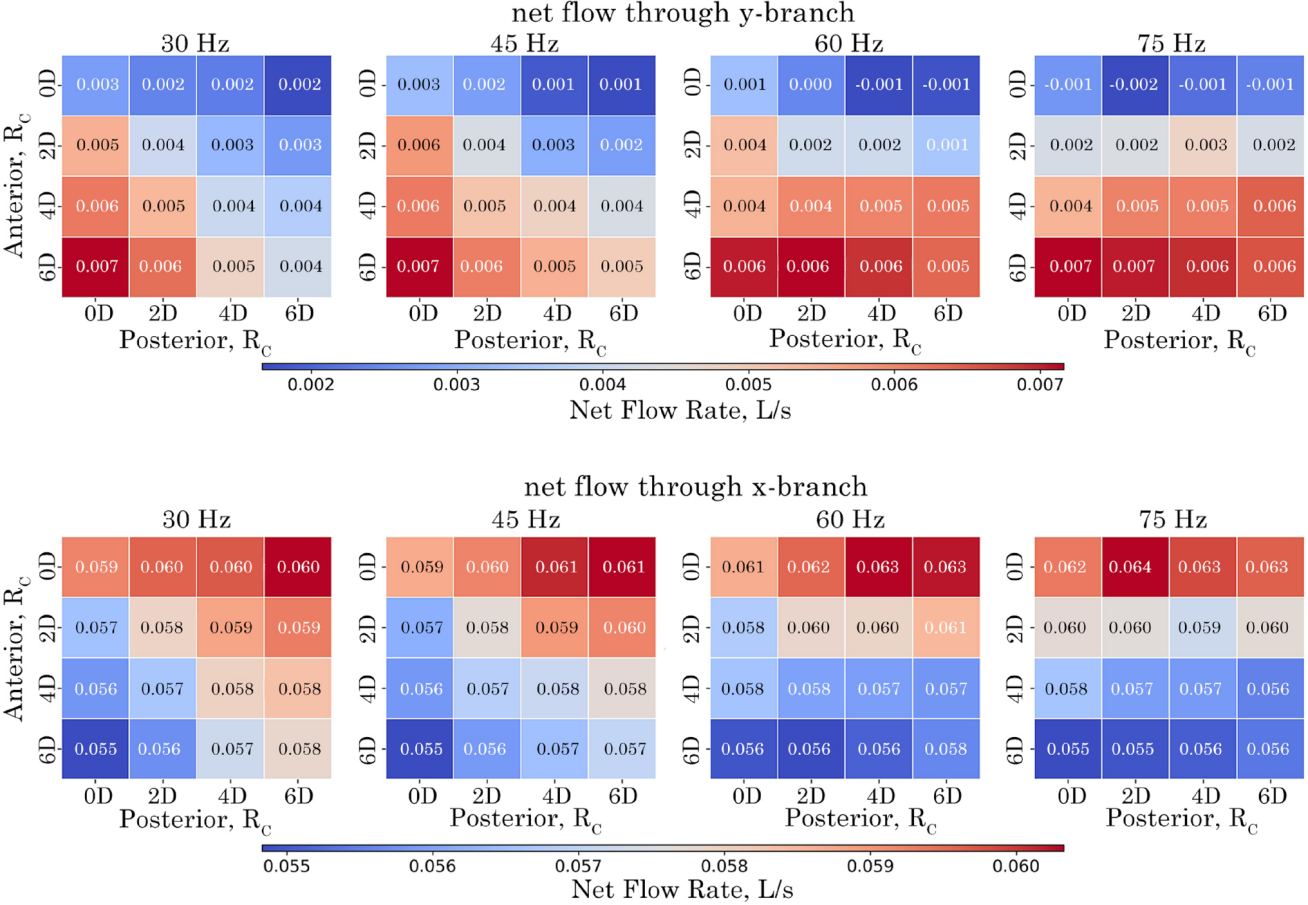
Net volumetric-flow rate at the *y*- and *x*-branch outlets for $R_c$ configurations over the analysed period of 0.0667 s corresponding to 2, 3, 4, and 5 cycles for the respective frequencies of 30 Hz, 45 Hz, 60 Hz and 75 Hz

The net flow for all model $R_c$ configurations is shown as volume flow rates through the exits, represented by heat maps in Fig. 14. For all configurations and frequencies of 30 Hz and 45 Hz, the net flow is positive (exiting the domain) at the *y*-branch outlet. However, a negative net flow occurs in models with an anterior $R_c$ of 0*D* as the frequency increases. At 60 Hz, negative flow appears when the posterior $R_c$ exceeds 4*D*. When the frequency reaches 75 Hz, all posterior models exhibit negative net flow due to the increased influence of the Bernoulli effect.

Increasing the anterior $R_c$ results in additional outflow, with a maximum of 0.007 L/s observed at an $R_c$ of 6*D* and a frequency of 30 Hz. Conversely, decreasing the anterior $R_c$ reduces the outflow magnitude. Similarly, increasing the posterior $R_c$ reduces outflow magnitude, but this effect weakens as frequency increases.

For the *x*-branch, the heat map shows an almost symmetric inverse relationship to the *y*-branch flow rate, due to mass conservation. Increasing the anterior $R_c$ decreases the net flow in the *x*-branch at all frequencies. In contrast, increasing the posterior $R_c$ increases the outflow in the *x*-branch. At 75 Hz, the net flow rates become independent of anterior and posterior changes, and no return flow is observed as the *x*-branch aligns with the bulk fluid flow.

**Fig. 15:**
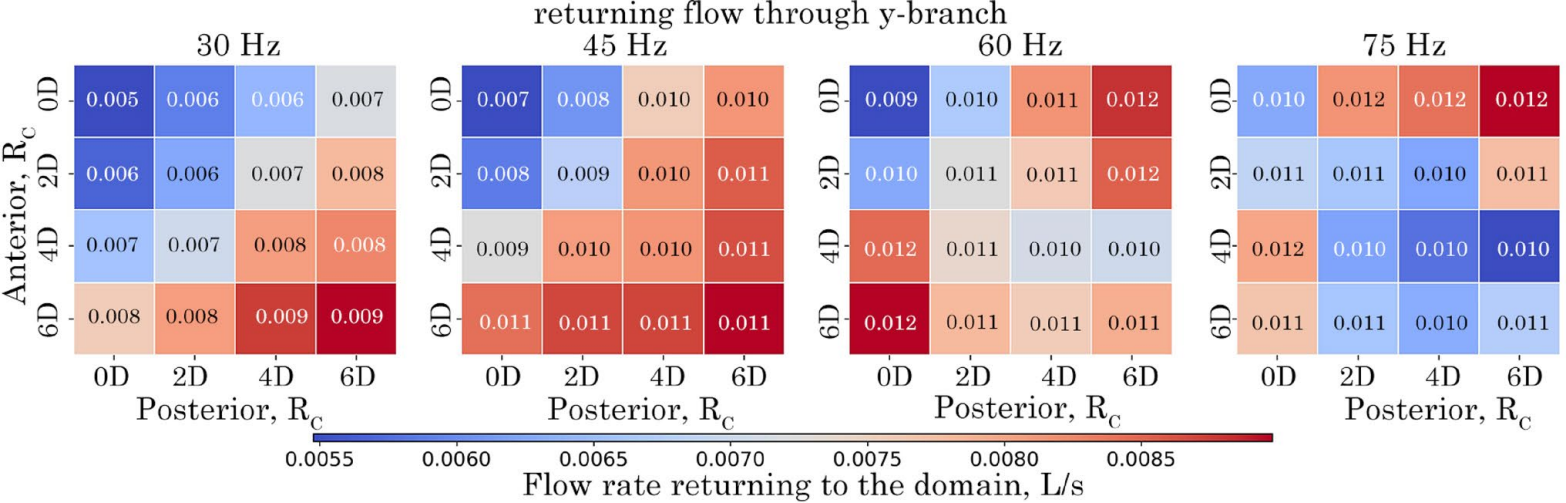
Amount of returning flow rate (suction) at the *y*-branch outlet for $R_c$ configurations over the analysed period of 0.0667 s corresponding to 2, 3, 4, and 5 cycles for the respective frequencies of 30 Hz, 45 Hz, 60 Hz and 75 Hz

The amount of returning flow (i.e. reverse, and suction) in the *y*-branch tube is shown in Fig. 15. As frequency increases the reverse flow in the *y*-branch also increases for all models. The maximum returning flow occurs when the anterior or posterior $R_c$ is increased to 6*D*. No returning flow was observed for the *x*-branch outlet.

**Fig. 16:**
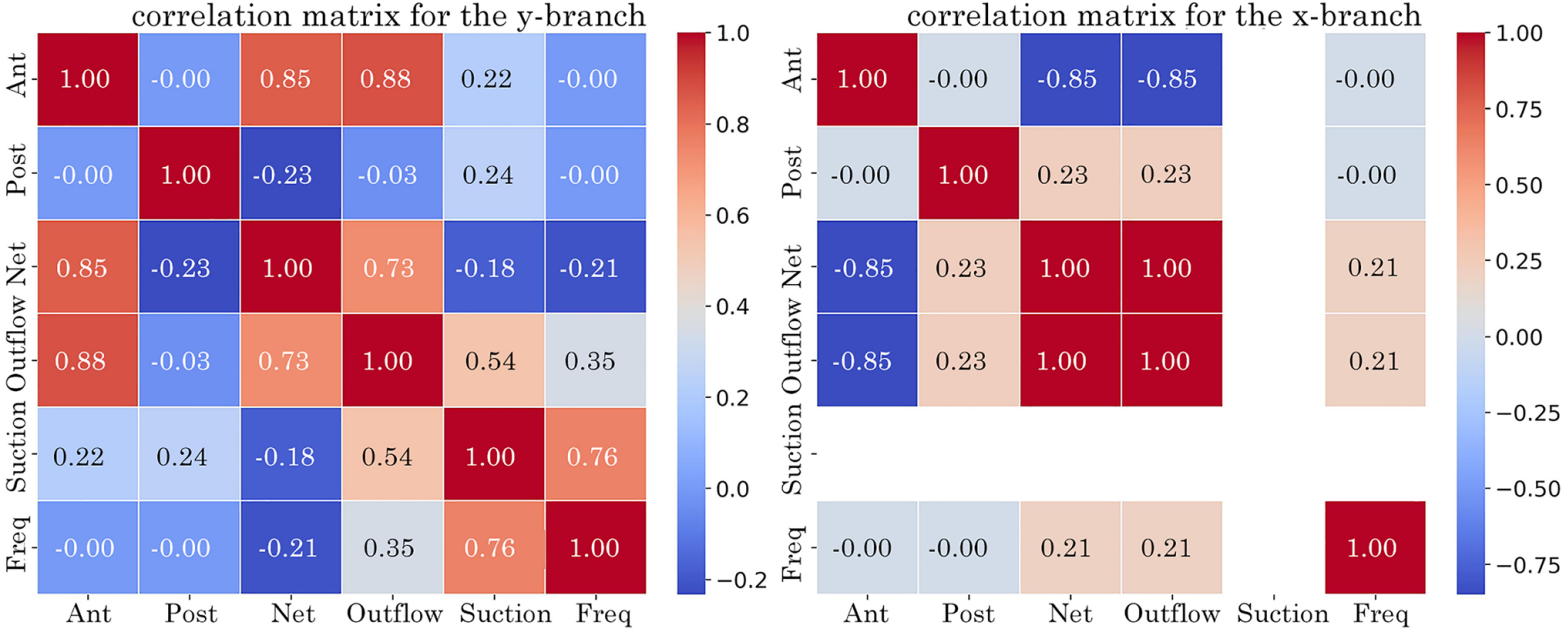
Correlation matrix at the *y*-branch and *x*-branch outlets identifying the relationship between analysed variables: frequency, suction (returning flow), outflow (exiting flow), net volumetric flow and $R_c$

The correlation matrix, in Fig. 16, highlights the strength of correlations between parameters. A value of 1 indicates a direct positive correlation, while a value of −1 suggests a direct inverse correlation. Three dominant effects are seen across the tubes. In the *y*-branch tube, there is a strong positive correlation (0.88) between the anterior $R_c$ and outflow, meaning that increasing the anterior $R_c$ leads to greater exit flow. There is also a strong positive correlation (0.76) between frequency and suction (reverse flow into the tube), indicating that higher frequency increases suction flow. In the outlet tube, there is a strong inverse correlation (−0.85) between the anterior $R_c$ and outflow, meaning that as the anterior $R_c$ increases, outflow decreases.

### Flow penetration and ventilation in nasal models

The volumetric-flow rate was recorded every 5 time steps at cross-sectional planes at the left and right maxillary ostium for both the Post-Op and Revision surgery models. As the Revision model maxillary ostia’s are significantly larger, the cross-section plane was divided into 4 quadrants with quadrants 1 and 2 representing the anterior zone and quadrants 3 and 4 representing the posterior zone.

**Fig. 17:**
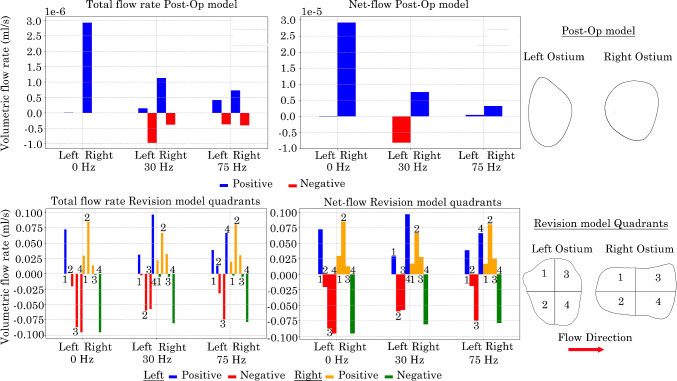
Positive and negative total volumetric-flow rates at left and right maxillary ostium in nasal models. Revision model presented as quadrants with the flow moving from left to right

The total positive, negative and net flow rate at the maxillary ostium was calculated by taking the positive and negative integrals between 0.0667$-$0.1333 s for both the Post-Op and Revision surgery models, Fig. 17. In the Post-Op model, the left ostium exhibited low volumetric-flow rates, with an increase in both penetration associated with increasing pulsating frequency. Both 30 Hz and 75 HZ induced suction at the left ostium due to the flow pulsation. Under 0 Hz and 75 Hz frequency conditions the left ostium experienced net positive volumetric-flow rates, indicating a tendency towards penetration. However under the 30 Hz condition suction was favoured with a net negative volumetric-flow rate. While the right ostium demonstrated higher positive flow rates compared with the left, it also showed significantly smaller net negative flow rates, suggesting enhanced penetration of the right maxillary ostium.

In the Revision model, the left ostium favoured negative flow rate in quadrants Q2, Q3 & Q4, and a positive flow rates in quadrant Q1 (see Fig. 17). The upper anterior quadrant (Q1) consistently exhibited a positive net volumetric-flow rate across all frequency conditions, whereas quadrants Q2 and Q3 remained negative for pulsating frequencies 0 Hz and 30 Hz. The lower posterior quadrant (Q4) exhibited net negative flow rates at both 0 Hz and 75 Hz conditions, it demonstrated a positive net flow rate during the 30 Hz pulsating flow condition. The right ostium exhibited a tendency towards positive net flow rates at the upper quadrants (Q1 & Q3) and lower anterior quadrant (Q2) under all frequency conditions. Conversely, a net negative flow rate was observed in the lower posterior quadrant (Q4).

### Normal velocity contours at maxillary ostium opening

**Fig. 18:**
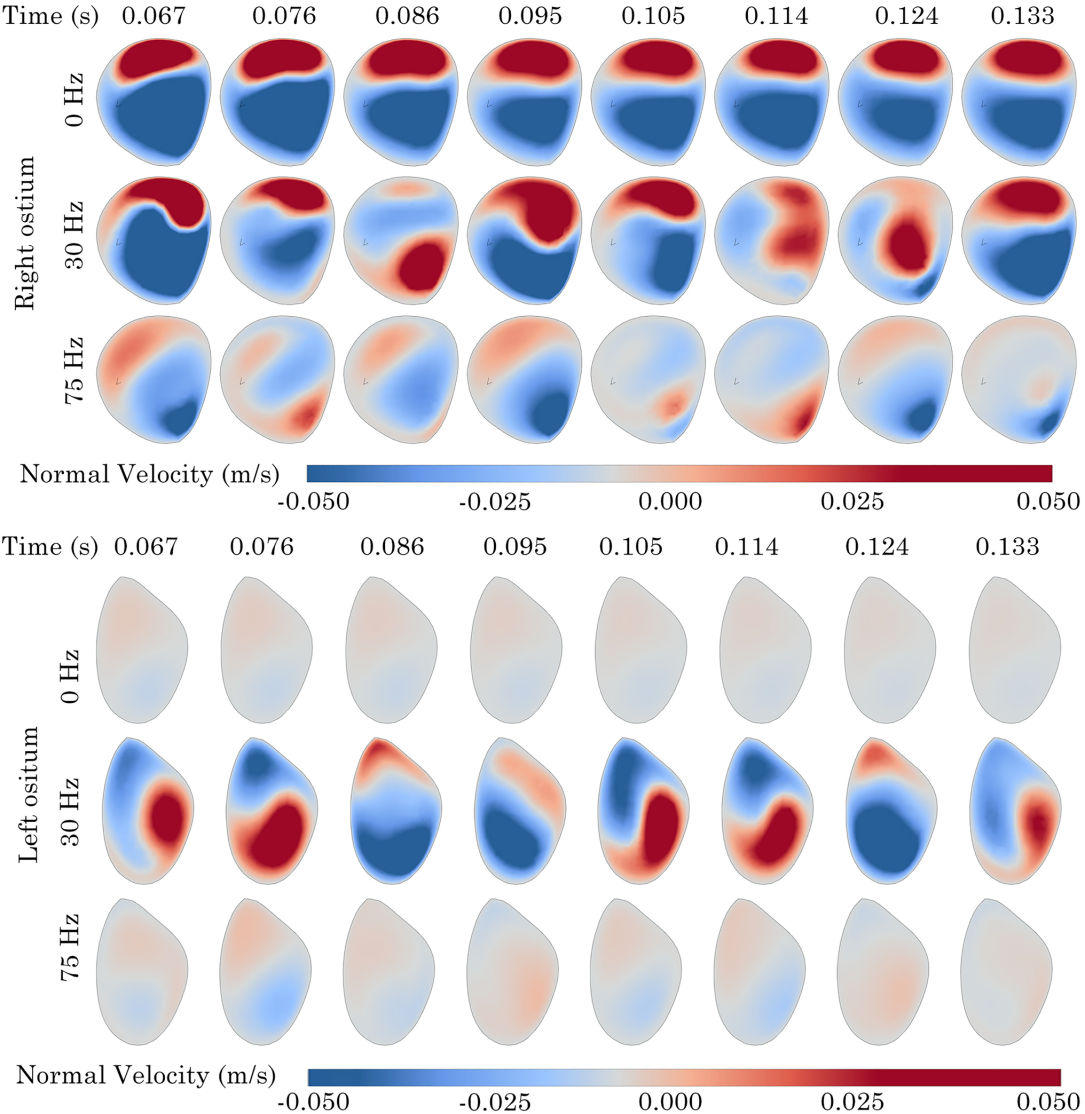
Normal velocity contours at left and right maxillary ostium on the Post-Op model. Positive values indicate sinus penetration

**Fig. 19:**
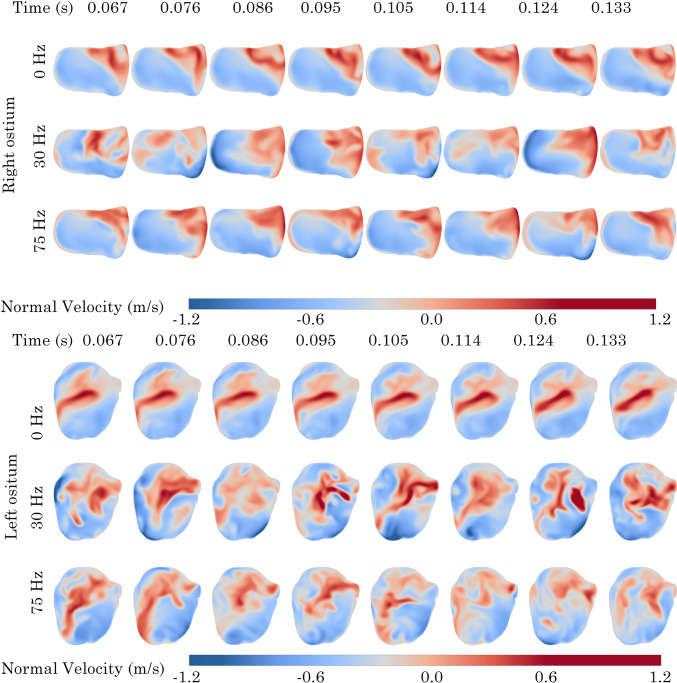
Normal velocity contours at left and right maxillary ostium on the Revision surgery model. Positive values indicate sinus penetration

The normal velocity fluctuations at the maxillary ostium were recorded over a time interval of 0.0667$-$0.1333 s to identify trends in air intake and expulsion from the maxillary sinus. Contours of normal velocity over 8 time points are presented in Figs. 18 & 19.

In the Post-Op model at 0 Hz, minimal fluctuations in normal velocity were observed at the right ostium. Conversely, the left ostium exhibited a clockwise rotating zone of positive velocity that moved from the lower anterior region to the upper area before dissipating. When the frequency was raised to 30 Hz, additional fluctuations in normal velocity appeared at the right ostium, with positive velocity noted on the posterior side. The left ostium showed a more pronounced and greater magnitude of positive velocity rotation. Increasing the frequency to 75 Hz further reduced the magnitude of velocity flux, Fig 18.

In the Revision model, comparable patterns in normal velocity corresponding to the net volumetric-flow rates, as shown in Fig. 17, were noted in each quadrant. At 0 Hz, the upper posterior zone (Q4) of the right ostium consistently exhibited positive normal velocity across all time points. In the left ostium, a centralised region of positive normal velocity was consistently observed across all time points. As the pulsating frequency increased, there was a broader distribution of positive velocity zones throughout the planes of both the left and right ostia, with a trend of positive normal velocity towards the upper posterior zone in the right ostium and a centralisation in the left ostium.

## Discussion

This study has practical implications for surgical procedures. It demonstrated the effect of anterior and posterior $R_c$ for a T-junction, a common geometrical configuration in many piping settings, which was used as a simplified representative model of the maxillary ostium. In general, a goal of FESS at the maxillary ostium is to widen the ostium diameter, to improve ventilation and restore mucociliary clearance (Anselmo-Lima et al. 2007; Hood et al. 2009; Xiong et al. 2008). Whilst computational studies analysing post-FESS models have reported enhanced simulated airflow by widening the ostium, they highlight that significant anatomy is removed to achieve this restoration that may lead to excess drying of the mucosal surfaces Frank et al. (2013); Xiong et al. (2008); Chen et al. (2011). Hood et al. (2009) found a correlation between the size of the maxillary ostium, volumetric-flow rate and velocity. Highlighting that as the ostium diameter is increased, fluid passes through at a higher velocity resulting in further drying.

This study performed a parametric analysis investigating the influence of potential surgical manoeuvres that open up the ostium. The enlargement size can play a significant role in airflow penetration, leading to potential improvements in patient recovery and comfort. The results aid in improving surgical decisions by providing insight into surgical planning through fluid dynamics behaviour through the maxillary ostium. Although drug particle transport was outside of the scope of this study, an oscillatory flow condition was used to represent a common drug delivery strategy of nebuliser or acoustic/oscillatory flow, to enhance flow penetration into the perpendicular-aligned ostium tube.

The choice of anterior and posterior $R_c$ represent surgical removal of bone and tissue that opens up the respective openings of the ostium. Siu et al. (2020) demonstrated flow streamlines entered the ostium posteriorly in patients with chronic rhinosinusitis, although this flow was biased by the non-perpendicular arrangement of the ostium tube. Thus, it is evident that introducing asymmetry to the ostium tube orientation may assist in flow penetration into the maxillary sinus. Our parametric study results found that increasing the anterior $R_c$ (i.e. surgically opening the anterior part of the ostium) enhances flow penetration into the maxillary sinuses, whereas modification of the posterior region had little effect. Although, modifying only the anterior region, rather than enlarging the entire diameter of the ostium, may restore mucociliary clearance whilst reducing post-FESS drying, this study has limited the assessment to pulsating frequencies $\ge$ 30 Hz and $\le$ 75 Hz and may not represent real-world applications. We did not test more extreme $R_c$ values past 6*D*, as this may not always be possible due to patient anatomical limitations.

The oscillatory frequency airflow affects the penetration and ventilation of the maxillary sinus. Higher oscillatory frequency results in additional suction at the maxillary ostium. While this would draw excess fluid from the maxillary sinus and aid in clearing excess mucous, it may also impede the delivery and penetration of inhaled drugs used to alleviate post-surgical symptoms. Although this study found comparable net fluid transfer at the maxillary sinus for frequencies of 30 Hz and 45 Hz, applying a frequency of 45 Hz offers increased exit and reverse flow from the sinus. Several studies investigating pulsating drug delivery devices have applied a standard frequency of 45 Hz, validating our finding (Granqvist et al. 2006; Laube 2007; Möller et al. 2010; Si et al. 2013; Xi et al. 2017). Although a frequency of 45 Hz is considered standard for nasal nebuliser devices, studies have found that higher frequencies can provide more than a twofold increase in drug deposition (Pourmehran et al. 2020; Xi et al. 2017; Leclerc et al. 2014; Durand et al. 2011).

Although the effect of oscillatory frequency on fluid penetration of the maxillary sinus has been well explored, this study found that the anterior $R_c$ has the greatest correlation to airflow penetrating the ostium.

The choice of $R_c$ and frequency is dependent on the desired outcome of the surgical procedure and post-surgical drug delivery. If the patient is experiencing excessive mucus blocking the ostium, applying higher oscillatory frequencies may help alleviate the blockage. However, reducing the oscillatory frequency could be more beneficial if the goal is to enhance drug penetration into the maxillary sinus. An *in vivo* study by Möller et al. (2010) found that larger ostium diameters (> 5 mm) had a negative effect on sinus ventilation when coupled with a pulsating flow frequency of 45 Hz. This finding suggests that traditional surgical methods of increasing the ostium diameter to enhance penetration may not be beneficial if the primary goal is to increase ventilation.

The results of this study indicate that anatomical parameters, specifically the $R_c$ and ostium size exert a greater influence on localised airflow dynamics than variations in pulsation frequency, within the constraints of the current model. It is important to note, however, that pulsation amplitude was held constant, and acoustic damping mechanisms were not incorporated. Consequently, the potential influence of resonance phenomena, as might occur in a Helmholtz-like system, remains unquantified. Future studies incorporating variable amplitude inputs and acoustic modelling are recommended to comprehensively evaluate the coupled effects of geometry and unsteady flow conditions on sinus ventilation. These findings underscore the potential for surgical modification to provide significant improvements in sinonasal airflow.

Although this study provided insight into ventilation and penetration of the maxillary ostium, it was limited to simple geometry configurations and two nasal cavities. As the human nasal cavity varies considerably between patients, it is recommended to use this study as a guide for further investigation.

## Conclusion

T-junctions are essential in engineering, used for fluid flow management, mixing, and distribution in industries like chemical processing and petroleum. This study investigates a circular T-junction with bifurcating flow, typical in industrial and biomedical applications. Building on prior research, it examines how factors such as pulsating flow and variations in the anterior and posterior $R_c$ affect airflow behaviour and flow division.

Our findings show that increasing the anterior $R_c$ significantly enhances flow penetration into the *y*-branch, while posterior curvature changes have a lesser impact. Higher oscillatory frequencies, particularly those above 45 Hz, increase reverse flow in the *y*-branch, potentially improving ventilation but for particle delivery may require a lower frequency. These insights are critical for applications like nasal drug delivery, where enhanced flow penetration into secondary passages is desired, and for systems requiring efficient phase separation or fluid distribution.

The results of this study emphasise the importance of T-junction geometry in optimising flow behaviour and suggest that modifications to the anterior $R_c$ are more effective than adjustments to the posterior curvature for enhancing flow penetration. While oscillatory frequency influences flow distribution, it is the geometry of the T-junction that plays a dominant role. These conclusions have broad implications, ranging from biomedical devices to industrial processes, where precise control of fluid distribution is essential. Future research could explore more extreme geometric modifications and their effects on multiphase flow, as well as the role of particle transport for applications like drug delivery and chemical processing.

Future work could explore more complex geometric modifications beyond the anterior and posterior $R_c$ tested in this study, such as introducing asymmetrical curvatures or varying the T-junction angle to further optimise flow behaviour and investigating pulsating amplitude. Additionally, extending the analysis to multiphase flows could provide insights relevant to industries where phase separation is critical. Incorporating particle tracking into the simulations would offer valuable data on particle deposition and transport, important for applications like nasal drug delivery and respiratory therapies. Finally, investigating the impact of higher frequencies and more extreme pulsating conditions could provide deeper understanding for optimising devices like nebulisers and industrial fluid systems.

This study highlighted that the choice of surgical operation has a large effect on the ventilation of the maxillary sinus. While the general approach of widening the maxillary ostium enhances fluid flow, it requires significant removal of nasal anatomy that has been associated with additional patient discomfort. By applying a $R_c$ at the anterior position of the maxillary ostium, ventilation is enhanced while minimising the amount of material removed. Applying a pulsating flow condition further enhanced ventilation and penetration into the maxillary sinus through the ostium length. We found that:

- Maximising the anterior $R_c$ at the maxillary ostium enhances both ventilation and penetration.
- Posterior $R_c$ has minimal effect on the flow field of the maxillary ostium.
- A pulsating flow frequency of $\sim$30 Hz will provide additional penetration and ventilation into the maxillary sinus.
- Increasing the pulsating frequency past 30 Hz may provide additional ventilation of the maxillary sinus.

## Data Availability

The data that support the findings of this study are available upon request.
